# Burmannic Acid Inhibits Proliferation and Induces Oxidative Stress Response of Oral Cancer Cells

**DOI:** 10.3390/antiox10101588

**Published:** 2021-10-10

**Authors:** Su-Ling Liu, Kun-Han Yang, Che-Wei Yang, Min-Yu Lee, Ya-Ting Chuang, Yan-Ning Chen, Fang-Rong Chang, Chung-Yi Chen, Hsueh-Wei Chang

**Affiliations:** 1 Experimental Forest College of Bioresources and Agriculture, National Taiwan University, Zhushan Township, Nantou County 55750, Taiwan; sulingliu@ntu.edu.tw; 2 Graduate Institute of Natural Products, Kaohsiung Medical University, Kaohsiung 80708, Taiwan; kunhan1013@gap.kmu.edu.tw (K.-H.Y.); u110531013@gap.kmu.edu.tw (C.-W.Y.); aaronfrc@kmu.edu.tw (F.-R.C.); 3 Department of Biomedical Science and Environmental Biology, College of Life Science, Kaohsiung Medical University, Kaohsiung 80708, Taiwan; u107023047@kmu.edu.tw (M.-Y.L.); u107023007@gap.kmu.edu.tw (Y.-T.C.); u107023010@gap.kmu.edu.tw (Y.-N.C.); 4 Department of Nutrition and Health Sciences, School of Medical and Health Sciences, Fooyin University, Kaohsiung 83102, Taiwan; 5 Center for Cancer Research, Kaohsiung Medical University, Kaohsiung 80708, Taiwan; 6 Department of Medical Research, Kaohsiung Medical University Hospital, Kaohsiung 80708, Taiwan

**Keywords:** burmannic acid, spices, oral cancer, DNA damage, apoptosis, oxidative stress

## Abstract

Burmannic acid (BURA) is a new apocarotenoid bioactive compound derived from Indonesian cinnamon; however, its anticancer effect has rarely been investigated in oral cancer cells. In this investigation, the consequences of the antiproliferation of oral cancer cells effected by BURA were evaluated. BURA selectively suppressed cell proliferation of oral cancer cells (Ca9-22 and CAL 27) but showed little cytotoxicity to normal oral cells (HGF-1). In terms of mechanism, BURA perturbed cell cycle distribution, upregulated mitochondrial superoxide, induced mitochondrial depolarization, triggered γH2AX and 8-hydroxy-2-deoxyguanosine DNA damage, and induced apoptosis and caspase 3/8/9 activation in oral cancer cells. Application of *N*-acetylcysteine confirmed oxidative stress as the critical factor in promoting antiproliferation, apoptosis, and DNA damage in oral cancer cells.

## 1. Introduction

There were 377,713 new cases and 177,757 deaths worldwide caused by oral cancer in 2020 [1]. For oral cancer, males have twice the incidence than females. Oral cancers cause exceptionally high morbidity in South Central Asia and Melanesia, contributing to the habit of betel nut chewing [1]. Oral squamous cell carcinomas comprise the majority of oral cancers (>90%) [2]. Oral cancer may grow on the tongue, cheeks, palate, lips, or gums. Several tumor markers have been reported [3], but they may display differential expression in different oral cancer locations [4]. Including more oral cancer cell types for drug discovery in oral cancer studies may provide wider potential applications to cure oral cancer. Chemo and radiotherapy for oral cancer is frequently accompanied by side effects [5]. Hence, it is important to discover more anticancer agents to solve this problem.

Several natural dietary products have anticancer effects [6,7]. Spices are also reported as potential anticancer sources [8,9,10,11,12]. For example, extracts of Indian spices can suppress the proliferation of esophageal cancer cells [9]. The “king of spices”, pepper or *Piper nigrum,* also shows anticancer effects [11]. The therapeutic effects of spices can be attributed to their bioactive compounds, including alkaloids, terpenes, flavonoids, phenylpropanoids, and anthocyanins [10].

Moreover, dietary phytochemicals have been shown to generate oxidative stress and to induce the killing of cancer cells [13]. Several spices also induce apoptosis of cancer cells as an anticancer effect. Therefore, the anticancer effect of different spices warrants detailed investigation, especially by study of the role of oxidative stress and apoptosis.

Many dietary isoprenoids, including β-ionone, exhibit chemo-preventive functions [14,15]. β-ionone demonstrated selective killing, anti-metastatic, and apoptosis-inducing abilities towards tumor cells in vitro and in vivo [16,17]. The endocyclic double bond in β-ionone undergoes epoxidation to 5,6-epoxy-β-ionone [18], which was more effective in inhibiting phorbol ester action in lymphocytes than β-ionone. Therefore, detailed investigation to identify the function of β-ionone derivatives is warranted.

*Cinnamomum burmannii*, also called Indonesian cinnamon, is widely distributed in Southeast Asia and Indonesia. *C. burmannii* is a commonly used spice in Indonesia [19], and its bark is the source of the spice cinnamon. Some homosesquiterpenoids [20] and amides have been isolated from *C. burmannii* stems. Using *C. burmannii* roots, we identified a new apocarotenoid and a novel β-ionone derivative, burmannic acid (BURA) [21], with a carboxylic acid group binding to C-5 of 3-hydroxy-5,6-epoxy-β-ionone. However, its molecular functioning has not yet been reported, according to a PubMed search carried out by the authors.

The present investigation evaluated the antiproliferation effects of *C.* *burmannii*-derived BURA on oral cancer cells for the first time. Oral cancer cell lines derived from gingival, tongue, and buccal mucosa tissues (Ca9-22, CAL 27, and OC-2) were selected for testing the cellular response to BURA. We further addressed the detailed mechanism for anti-oral-cancer cell effects of BURA, focusing on oxidative stress, apoptosis, and DNA damage.

## 2. Materials and Methods

### 2.1. BURA Preparation

BURA (MW 296) was purified from the roots of *C. burmannii* as previously described [21]. Broadly, the roots of *C. burmannii* (203.4 g) were air-dried for MeOH (1 L × 3) extraction. After reduced pressure, the concentrated MeOH extract (11.2 g) was processed in a silica gel column for CH_2_Cl_2_ elution, to which MeOH was gradually added to generate three fractions. Part of fraction 3 (2.51 g) was processed by chromatography by n-hexane/EtOAc (100:1) elution, enriched with EtOAc, to generate four additional fractions (3-1~3-4). Fraction 3-1 (0.82 g) was re-processed by chromatography and purified by TLC analysis using n-hexane/EtOAc to yield BURA. The purity of BURA was greater than 90%, as confirmed by HPLC.

### 2.2. Reagents

To evaluate the involvement of oxidative stress, a specific inhibitor, *N*-acetylcysteine (NAC) [22,23,24], was applied in the pretreatments (10 mM, 1 h). It was purchased from Sigma-Aldrich (St. Louis, MO, USA) and dissolved in PBS. To evaluate the involvement of caspases, (Cas) 8 and 9, their specific inhibitors, such as Z-IETD-FMK (Z-IETD) and Z-LEHD-FMK (Z-LEHD) (Selleckchem.com; Houston, TX, USA), were applied in pretreatments (10 μM for 2 h) to examine the response to Cas 3 activity.

### 2.3. Cell Cultures

ATCC oral cancer (CAL 27) and normal oral (HGF-1) cell lines (Manassas, VA, USA), as well as RIKEN BioResource Research Center oral cancer (Ca9-22) cell lines (Tsukuba, Ibaraki, Japan), were used. The OC-2 oral cancer cell line was provided by Dr. Wan-Chi Tsai (Kaohsiung Medical University, Kaohsiung, Taiwan) [25]. The medium for cell culture of the CAL 27, Ca9-22, and OC-2 cells was a 3:2 mixture of Dulbecco’s Modified Eagle Medium (DMEM) and F12 (Gibco, Grand Island, NY, USA), while a 4:1 mixture was used for the HGF-1 cells. Other cell culture information has previously been reported [26].

### 2.4. Cell Viability Assay

A tetrazolium agent, able to sensitively react with mitochondrial enzymes in living cells, was used to generate colored formazan dye for detection at 490 nm by a multi-plate reader. A tetrazolium-based MTS kit (Promega Corporation, Madison, WI, USA) was employed, according to the user manual, to determine cell viability after 24 h of drug treatment [27].

### 2.5. Cell Cycle Determination

After overnight fixation with 75% ethanol, DNA was stained by 7-aminoactinmycin D (7AAD) (1 μg/mL, 30 min) (Biotium; Hayward, CA, USA) [28]. This processing enables cell cycle discrimination using an Accuri C6 flow cytometer (Becton-Dickinson, Mansfield, MA, USA).

### 2.6. Apoptosis Determination by Annexin V/7AAD Method

Apoptotic cells were detected using annexin V (1:1000 dilution)/7AAD (1 μg/mL) dyes [29] (Strong Biotech; Taipei, Taiwan), with binding buffers provided in the kit, for 1 h at 37 °C. The dye intensities were examined by flow cytometer (Accuri C6), to identify annexin V (+)/7AAD (+ or −) marked populations according to their apoptotic state.

### 2.7. Apoptosis Determination by Pancaspase and Caspase 3/8/9 (Cas 3/8/9) Assays

Caspase activation is a standard indicator for apoptosis signaling. To explore this characteristic of apoptosis activity, several caspase-based detection assays were performed, including pancaspase- and Cas 3/8/9-detected flow cytometry. A pancaspase (active)-FITC staining kit (Abcam, Cambridge, UK) was used for the fast-screening of caspases-1 and 3 to 9 [27], according to the user’s manual. The pancaspase intensity was measured using a flow cytometer (Accuri C6).

In particular, the Cas 3/8/9 activities were individually examined by peptide-based flow cytometry [30]. In brief, substrates for detecting Cas 3/8/9 activities provided in OncoImmunin kits (Gaithersburg, MD, USA) (PhiPhiLux-G1D2, CaspaLux8-L1D2, and CaspaLux9-M1D2) are composed of a peptide, homo-doubly labeled with a fluorophore. The activated Cas 3/8/9 can cleave their specific substrates and generate green fluorescence for flow cytometry detection [30]. Cells were incubated with 10 μM substrate solution for Cas 3/8/9 (1:1000) at 37 °C for 1 h. After washing, their intensities were measured by a flow cytometer (Accuri C6) (FL1 channel). The flow cytometry for Cas 3/8/9 detections had to be performed separately because they show the same green fluorescence.

### 2.8. Oxidative Stress Determination by Mitochondrial Superoxide (MitoSOX), and Mitochondrial Membrane Potential (MMP) Assays

When oxidative stress was over-produced, MitoSOX generation and MMP destruction were induced. These were probed using MitoSOX™ Red [31] (50 nM, 30 min) and DiOC_2_(3) [32] (Invitrogen; San Diego, CA, USA) (5 nM, 30 min), respectively. These reactions were performed in darkness at 37 °C. Their intensities were measured by a flow cytometer (Accuri C6).

### 2.9. DNA Damage Determination by γH2AX and 8-Hydroxy-2-Deoxyguanosine (8-OHdG) Assays

DNA damage was detected by probing with the markers γH2AX [33] and 8-OHdG [34]. Briefly, cells were fixed overnight. After washing with PBS, γH2AX was detected by γH2AX antibody [33] (Santa Cruz Biotechnology; Santa Cruz, CA, USA) (4 °C, 1 h) and Alexa Fluor^®^488-labeled secondary antibody (Cell Signaling Technology). Subsequently, 7AAD (5 μg/mL, 30 min) was added. 8-OHdG was detected by 8-OHdG-FITC antibody (Santa Cruz Biotechnology) (4 °C, 1 h). The γH2AX and 8-OHdG intensities were examined by flow cytometry (Accuri C6).

### 2.10. Statistical Analysis

The statistical significances of multi-comparisons were evaluated by one-way analysis of variance (ANOVA) followed by Tukey HSD post hoc test. Data top-labeled with different lower-case letters differ significantly.

## 3. Results

### 3.1. BURA Shows Selective Antiproliferation in Oral Cancer Cells

**Figure 1:**
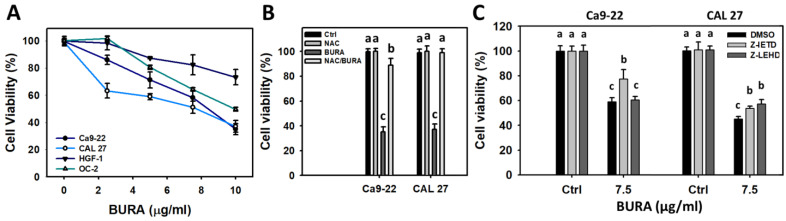
Cell viabilities of oral cancer cells following BURA exposure. (**A**) 24 h MTS assay. (**B**) NAC effects on cell viability of BURA-treated oral cancer cells. After NAC (10 mM, 1 h) treatments or not, oral cancer cells (Ca9-22, CAL 27, and OC-2) and normal oral cells (HGF-1) were treated with BURA (0 (control) and 10 μg/mL (33.8 μM)) for 24 h. NAC/BURA indicate the pre-and post-treatment. (**C**) Effects of Cas 8 and Cas 9 inhibitors on cell viability of BURA-treated oral cancer cells. After Cas 8 and Cas 9 inhibitor treatments (10 μM, 2 h), cells were treated with BURA (7.5 μg/mL (25.3 μM)) for 0 (control) for 24 h. Data, mean ± SD (*n* = 3). Data top-labeled with non-overlapping lower-case letters differ significantly with respect to multi-comparisons of the same cell line (*p* < 0.05).

The cell viability (%) of the oral cancer cells (Ca9-22, CAL 27, and OC-2) was suppressed by BURA in a dose-dependent manner (Figure 1A). In contrast, normal oral cells (HGF-1) maintained higher viability than the three oral cancer cell lines. Therefore, BURA can selectively inhibit proliferation of oral cancer cells but exhibits less cytotoxicity to normal oral cells.

To test oxidative stress involvement, an inhibitor (NAC) was applied to examine the change in cell viability of the oral cancer cells. The BURA-induced antiproliferation in oral cancer cells was alleviated by NAC pretreatment (Figure 1B). To test extrinsic (Cas 8) and intrinsic (Cas 9) apoptosis involvement, their inhibitors (Z-IETD and Z-LEHD) were applied to examine the change in the cell viability of the oral cancer cells. The BURA-induced antiproliferation of oral cancer cells was alleviated by Z-IETD pretreatment for Ca9-22 and CAL 27 cells (Figure 1C). The BURA-induced antiproliferation of oral cancer cells was alleviated by Z-LEHD pretreatment for CAL 27 cells but not for Ca9-22 cells.

### 3.2. BURA Induces Cell Cycle Redistribution in Oral Cancer Cells

**Figure 2:**
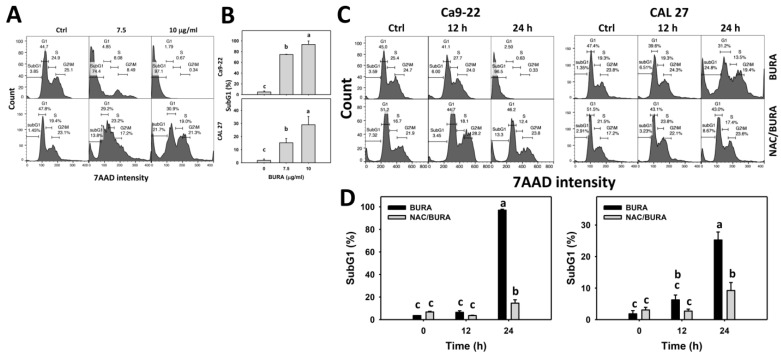
BURA causes cell cycle redistribution of oral cancer cells. (**A**,**B**) Cell cycle pattern and quantification. Oral cancer cells (Ca9-22 and CAL 27) were treated with BURA (control (0.1% DMSO), 7.5 μg/mL (25.3 μM), and 10 μg/mL (33.8 μM), 24 h). (**C**,**D**) NAC effect on cell cycle distribution and quantification. After NAC treatment (10 mM, 1 h), cells were treated with BURA (10 μg/mL) for 0 (control), 12 and 24 h. They were labeled with NAC and NAC/BURA. Data, mean ± SD (*n* = 3). Data top-labeled with non-overlapping lower-case letters differ significantly according to multi-comparisons of the same cell cycle phase (*p* < 0.05).

Antiproliferation is commonly associated with cell cycle redistribution [35,36]. Accordingly, the cell cycle changes in oral cancer cells following BURA treatment were monitored (Figure 2A); subG1 populations were more evident in BURA-treated oral cancer cells than in the controls (Figure 2B), suggesting that BURA causes subG1 accumulation, which is an apoptosis-indicating phenomenon.

In addition, the action of oxidative stress in cell cycle change was examined by NAC pretreatment (Figure 2C). The subG1 populations were more evident in BURA-treated oral cancer cells than in the controls, an effect which was reduced by NAC pretreatment (Figure 2D).

### 3.3. BURA Increases Annexin V-Positive Population in Oral Cancer Cells

**Figure 3:**
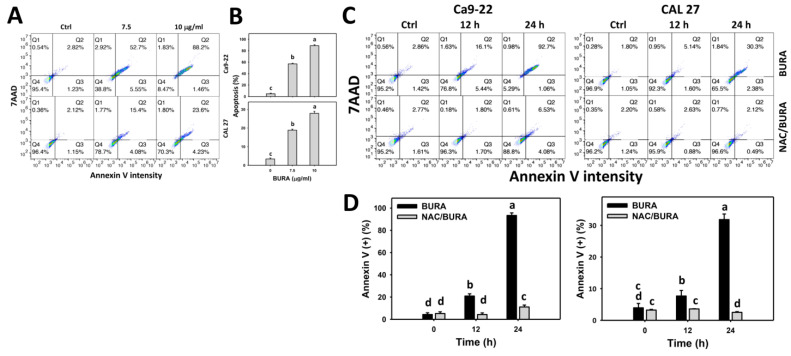
BURA causes apoptosis in oral cancer cells. (**A**,**B**) Annexin V pattern and quantification. Oral cancer cells (Ca9-22 and CAL 27) were treated with BURA (control (0.1% DMSO), 7.5 μg/mL (25.3 μM), and 10 μg/mL (33.8 μM), 24 h). Annexin V (+)/7AAD (+/−) populations (%) were counted to apoptosis (%). (**C**,**D**) NAC effect on annexin V pattern and quantification. After NAC treatment (10 mM, 1 h), cells were treated with BURA (10 μg/mL) for 0 (control), 12 and 24 h. Data, mean ± SD (*n* = 3). Data top-labeled with non-overlapping lower-case letters differ significantly according to multi-comparisons (*p* < 0.05).

Annexin V is a probe for detecting apoptotic cells. BURA-induced apoptosis of oral cancer cells was monitored by flow cytometry detection for annexin V/7AAD (Figure 3A). Annexin V (+) populations were more evident in BURA-treated oral cancer cells than in the controls in a dose-responsive manner (Figure 3B), indicating that BURA causes apoptosis.

Further, the action of oxidative stress in triggering apoptosis was examined for NAC pretreatment (Figure 3C). Annexin V (+) populations were more evident in BURA-treated oral cancer cells than in the controls in a time-dependent manner, which was reduced by NAC pretreatment (Figure 3D).

### 3.4. BURA Promotes Pancaspase Activation in Oral Cancer Cells

**Figure 4:**
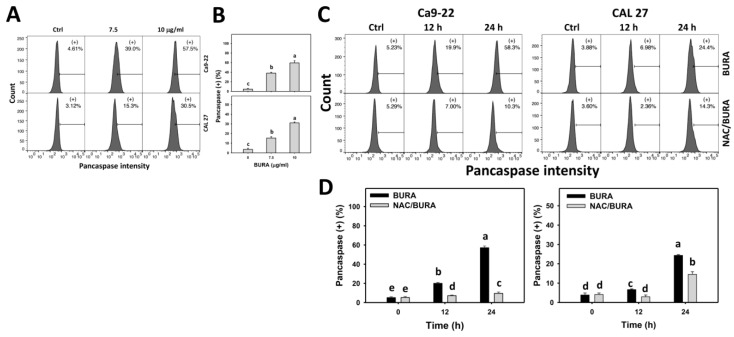
BURA causes pancaspase activation in oral cancer cells. (**A**,**B**) Pancaspase pattern and quantification. Oral cancer cells (Ca9-22 and CAL 27) were treated with BURA (control (0.1% DMSO), 7.5 μg/mL (25.3 μM), and 10 μg/mL (33.8 μM), 24 h). (+) located within the patterns represent pancaspase (+). (**C**,**D**) NAC effect on pancaspase pattern and quantification. After NAC treatment (10 mM, 1 h), cells were treated with BURA (10 μg/mL) for 0 (control), 12 and 24 h. Data, mean ± SD (*n* = 3). Data top-labeled with non-overlapping lower-case letters differ significantly according to multi-comparisons (*p* < 0.05).

Sequential activation of caspases is essential in triggering apoptosis [37]. BURA-induced apoptosis of oral cancer cells was monitored by flow cytometry detection for active pancaspase (Figure 4A). Active pancaspase (+) populations were more evident in BURA-treated oral cancer cells than in the controls in a dose-responsive manner (Figure 4B), indicating that BURA causes pancaspase activation triggering apoptosis.

Moreover, the role of oxidative stress in triggering pancaspase activation was examined by NAC pretreatment (Figure 4C). Active pancaspase populations were more evident in BURA-treated oral cancer cells than in the controls in a time-dependent manner, which was reduced by NAC pretreatment (Figure 4D).

### 3.5. BURA Promotes Caspase Signaling Activation in Oral Cancer Cells

**Figure 5:**
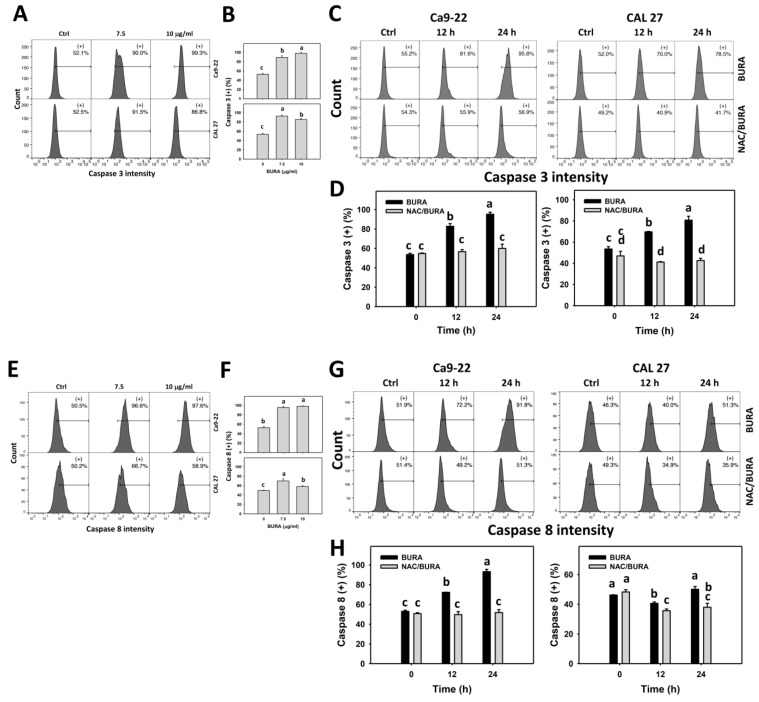

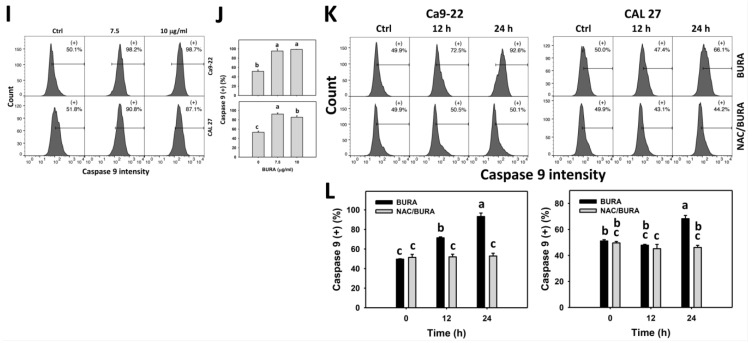
BURA causes caspase 3/8/9 activations in oral cancer cells. (**A**,**B**,**E**,**F**,**I**,**J**) Caspase 3/8/9 patterns and quantifications. Oral cancer cells (Ca9-22 and CAL 27) were treated with BURA (control (0.1% DMSO), 7.5 μg/mL (25.3 μM), and 10 μg/mL (33.8 μM), 24 h). (+) located within the patterns represent pancaspase (+). (**C**,**D**,**G**,**H**,**K**,**L**) NAC effect on Cas 3/8/9 patterns and quantifications. After NAC treatment (10 mM, 1 h), cells were treated with BURA (10 μg/mL) for 0 (control), 12 and 24 h. Data, mean ± SD (*n* = 3). Data top-labeled with non-overlapping lower-case letters differ significantly according to multi-comparisons (*p* < 0.05).

Since the active pancaspase kit detects some caspases, Cas 3/8/9 activations of apoptosis signaling were further assessed (Figure 5A,C,E,G,I,K). In Cas 3/8/9 flow cytometry, BURA promoted Cas 3/8/9 activations in oral cancer cells (Figure 5B,F,J). Moreover, these intrinsic (Cas 9) and extrinsic (Cas 8) apoptosis protein expressions were inhibited by NAC pretreatment (Figure 5D,H,L).

### 3.6. BURA Generates MitoSOX in Oral Cancer Cells

**Figure 6:**
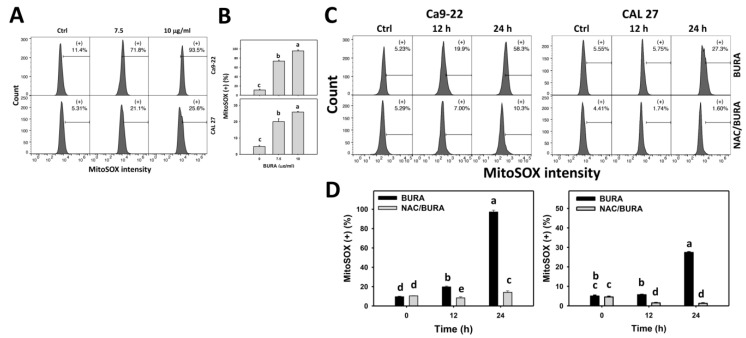
BURA causes MitoSOX production in oral cancer cells. (**A**,**B**) MitoSOX pattern and quantification. Oral cancer cells (Ca9-22 and CAL 27) were treated with BURA (control (0.1% DMSO), 7.5 μg/mL (25.3 μM), and 10 μg/mL (33.8 μM), 24 h). (+) located within the patterns represent MitoSOX (+). (**C**,**D**) NAC effect on MMP pattern and quantification. After NAC treatment (10 mM, 1 h), cells were treated with BURA (10 μg/mL) for 0 (control), 12 and 24 h. Data, mean ± SD (*n* = 3). Data top-labeled with non-overlapping lower-case letters differ significantly according to multi-comparisons (*p* < 0.05).

Oxidative stress, such as MitoSOX, was examined in the present study. It was monitored by flow-cytometry-based-MitoSOX detection (Figure 6A). MitoSOX (+) populations were more evident in BURA-treated oral cancer cells than in the controls in a dose-responsive manner (Figure 6B), suggesting that BURA causes oxidative stress.

Moreover, the action of oxidative stress in generating MitoSOX was examined by NAC pretreatment (Figure 6C). MitoSOX (+) populations were more pronounced in BURA-treated oral cancer cells than in the controls in a time-dependent manner, which was reduced by NAC pretreatment (Figure 6D).

### 3.7. BURA Causes MMP Reduction in Oral Cancer Cells

**Figure 7:**
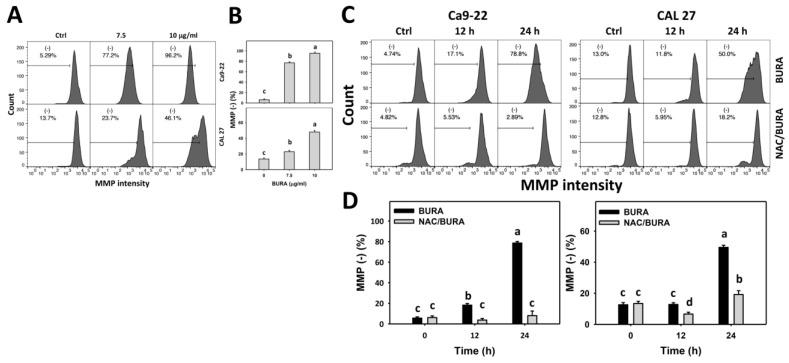
BURA causes MMP reduction in oral cancer cells. (**A**,**B**) MMP patterns and quantification. Oral cancer cells (Ca9-22 and CAL 27) were treated with BURA (control (0.1% DMSO), 7.5 μg/mL (25.3 μM), and 10 μg/mL (33.8 μM), 24 h). (−) located within the patterns represent MMP (−). (**C**,**D**) NAC effect on MMP pattern and quantification. After NAC treatment (10 mM, 1 h), cells were treated with BURA (10 μg/mL) for 0 (control), 12 and 24 h. Data, mean ± SD (*n* = 3). Data top-labeled with non-overlapping lower-case letters differ significantly for multi-comparisons (*p* < 0.05).

Mitochondrial dysfunction such as MMP reduction may induce oxidative stress and cell death [38]. Accordingly, change in MMP levels in oral cancer cells following BURA treatment was monitored (Figure 7A). MMP (−) populations were more evident in BURA-treated oral cancer cells than in the controls in a dose-responsive manner (Figure 7B), suggesting that BURA causes MMP reduction.

Moreover, the action of oxidative stress in MMP reduction was inspected by NAC pretreatment (Figure 7C). MMP (−) populations were more evident in BURA-treated oral cancer cells than in the controls in a time-dependent manner (Figure 7B), which was reduced by NAC pretreatment (Figure 7D).

### 3.8. BURA Causes DNA Damage in Oral Cancer Cells

**Figure 8:**
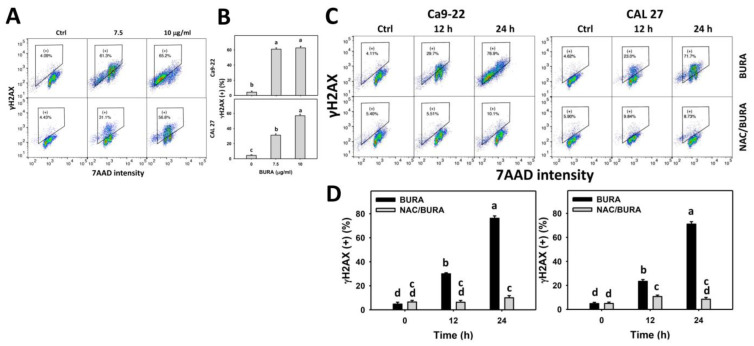
BURA causes γH2AX DNA damage in oral cancer cells. (**A**,**B**) γH2AX pattern and quantification. Oral cancer cells (Ca9-22 and CAL 27) were treated with BURA (control (0.1% DMSO), 7.5 μg/mL (25.3 μM), and 10 μg/mL (33.8 μM), 24 h). (+) located within the patterns represented by γH2AX (+). (**C**,**D**) NAC effect on γH2AX patterns and quantification. After NAC treatment (10 mM, 1 h), cells were treated with BURA (10 μg/mL) for 0 (control), 12 and 24 h. Data, mean ± SD (*n* = 3). Data top-labeled with non-overlapping lower-case letters differ significantly according to multi-comparisons (*p* < 0.05).

**Figure 9:**
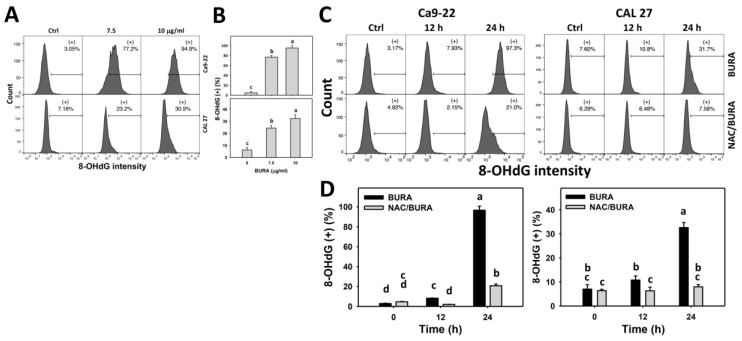
BURA causes 8-OHdG DNA damage in oral cancer cells. (**A**,**B**) 8-OHdG pattern and quantification. Oral cancer cells (Ca9-22 and CAL 27) were treated with BURA (control (0.1% DMSO), 7.5 μg/mL (25.3 μM), and 10 μg/mL (33.8 μM), 24 h). (+) located within the patterns represent 8-OHdG (+). (**C**,**D**) NAC effect on 8-OHdG patterns and quantification. After NAC treatment (10 mM, 1 h), cells were treated with BURA (10 μg/mL) for 0 (control), 12 and 24 h. Data, mean ± SD (*n* = 3). Data top-labeled with non-overlapping lower-case letters differ significantly according to multi-comparisons (*p* < 0.05).

DNA damage is also a typical response to oxidative stress [39]. BURA-induced DNA damage in oral cancer cells was monitored by flow cytometry-based γH2AX and 8-OHdG detection (Figure 8A and Figure 9A). γH2AX and 8-OHdG (+) populations were more evident in BURA-exposed oral cancer cells than in the controls in a dose-responsive manner (Figure 8B and Figure 9B), suggesting that BURA caused DNA damage.

Moreover, the action of oxidative stress in triggering DNA damage was examined by NAC pretreatment (Figure 8C and Figure 9C). γH2AX and 8-OHdG (+) populations were more evident in BURA-treated oral cancer cells than in the controls in a time-dependent manner, which was reduced by NAC pretreatment (Figure 8D and Figure 9D).

## 4. Discussion

The molecular functions of BURA have not previously been reported. In the present investigation, we validated, for the first time, the antiproliferation effects of *C. burmannii*-derived BURA on oral cancer cells. In terms of mechanism, BURA causes oxidative stress involving cell cycle disturbance, apoptosis, and DNA damage in oral cancer cells.

### 4.1. BURA Shows Antiproliferation Effect in Oral Cancer Cells

In the present study, the *C. burmannii* root-derived BURA showed IC_50_ values of 8.1, 7.5, and 10 µg/mL (27.4, 25.3, and 33.7 µM) at a 24 h MTS assay of oral cancer cells (Ca9-22, CAL 27, and OC-2). In contrast, BURA showed less cytotoxicity to normal oral cells than to oral cancer cells. Since cytotoxicity for BURA was not identified in a PubMed search (2021/07/30), compounds from the stems of *C. burmannii,* such as β-sitosterol [20], were compared. β-sitosterol showed an IC_50_ value of 75 and 266.2 µM at a 24 h MTT assay in lung (A549) [40] and colon (COLO 320) [41] cancer cells. By comparison, the clinically applied drug cisplatin showed an IC_50_ value of 7.9 and 9.6 µM (MW 301.1) at a 24 h MTS assay in oral cancer cells (Ca9-22 and HSC-3) [34]. In a 72 h MTS assay, cisplatin-treated oral cancer cells (SAS and H103) showed IC_50_ values of 3.74 and 20.12 µM, respectively [42]. Therefore, the antiproliferation effect of BURA in oral cancer cells has first been reported in the present study.

### 4.2. BURA Shows Oxidative Stress in Oral Cancer Cells

Many cancers exhibit oxidative stress overproduction and mitochondrial dysfunction, resulting in cellular oxidative damage [43]. Drugs with oxidative stress accumulation ability may destroy redox homeostasis to kill cancer cells and become welcome chemical additions in anticancer therapy [44,45]. For example, pomegranate extract (POMx) [7], manoalide [26], sinuleptolide [31], cryptocaryone [32], 4beta-hydroxywithanolide E [33], and tenuifolide B [46], can generate oxidative stress and trigger apoptosis in oral cancer cells.

Mitochondria are the primary source of oxidative stress because of electron leakage during ATP production. Although an antioxidant system is available in mitochondria, drug-induced ROS overexpression may exceed the tolerance of this defense system, causing mitochondrial damage [38]. This ROS overexpression may trigger MitoMP depolarization for MitoMP loss. Consequently, damaged mitochondria generate more oxidative stress, such as ROS and MitoSOX [47]. Therefore, drug-induced minor MitoMP loss may be amplified by this positive feedback action to induce cell death. Moreover, drug-induced oxidative stress may cause several types of non-apoptosis cell death in addition to apoptosis [48]. Apart from MitoMP loss, other lethal processes may be dysregulated by the drug-induced oxidative stress response, including autophagy, ferroptosis, and necroptosis.

Similarly, BURA demonstrated MitoSOX overproduction and MMP reduction in oral cancer cells (Figure 6 and Figure 7), revealing that BURA exhibits oxidative stress-regulated functions in oral cancer cells. Moreover, these oxidative stresses may be accompanied by the activation of compensatory antioxidant signaling [43]. This warrants detailed investigation of the antioxidant gene responses for oral cancer cells following BURA treatment.

In addition, drug-induced oxidative stress may regulate the Warburg effect in cancer cell proliferation. For example, the red ginseng-derived ginsenoside, 20(S)-Rg3, suppressed the Warburg effect by inhibiting the proliferation of ovarian cancer cells [49]. Morin inhibited the Warburg effect and induced apoptosis, to cause antiproliferation in colon cancer cells [50]. Similarly, BURA induced oxidative stress in oral cancer cells. This warrants a detailed examination of the Warburg effect in oral cancer cells following BURA treatment in the future.

### 4.3. BURA Shows Apoptosis and DNA Damage in Oral Cancer Cells

Oxidative stress overproduction can also induce apoptosis and become an effective anticancer strategy [51]. Moreover, oxidative stress accumulation also causes cell cycle disturbance and triggers caspase signaling leading to apoptosis [52]. For example, tenuifolide B from *C. tenuifolium* overproduces oxidative stress and triggers apoptosis in oral cancer cells [46]. Similarly, BURA induces subG1 accumulation (Figure 2) and annexin V-detected apoptosis in oral cancer cells (Figure 3). The involvement of caspase was confirmed by pancaspase activation (Figure 4). More specifically, BURA activates the end executors of apoptosis, such as Cas 3 (Figure 5). The extrinsic and intrinsic apoptosis proteins, such as Cas 8 and Cas 9, were also activated by BURA. In addition, the cell death contributions of Cas 8 and Cas 9 were examined by using their inhibitors (Figure 1C). For Ca9-22 cells, Cas 8 had higher killing effects on cell viability after BURA treatment than Cas 9. For CAL 27 cells, both Cas 8 and Cas 9 have killing effects on cell viability after BURA treatment. Therefore, Cas 8 and Cas 9 may involve differential regulatory processes for the viability of different oral cancer cell types.

Furthermore, high oxidative stress levels can damage macromolecules such as proteins, lipids, and DNA, and destroy their functions [52]. For DNA damage, oxidative stress may induce oxidative changes, such as double-strand breaks and oxidative binding to nucleotides such as γH2AX and 8-OHdG, respectively [52]. Consistent with the present study, γH2AX and 8-OHdG were highly expressed in oral cancer following BURA treatments. In addition to DNA damage, oxidative stress also causes protein damage by peroxidation that may suppress DNA repair ability. For example, UVA promotes oxidative stress and promotes DNA repair inhibition [53]. This warrants specific inspection of DNA repair responses in oral cancer cells following BURA treatment.

### 4.4. Antiproliferation Mechanisms of BURA in Oral Cancer Cells Depend on Oxidative Stress

NAC is a common antioxidant for oxidative stress removal in several oxidative stress studies. The action of oxidative stress after drug-induced changes was confirmed by NAC pretreatment [54]. For example, manoalide promotes antiproliferation, apoptosis, and DNA damage, and these changes are suppressed by NAC [26]. Similarly, NAC reduces antiproliferation, reverses the cell cycle disturbance and apoptosis, and suppresses the DNA damage for BURA-treated oral cancer cells. These findings support the view that the antiproliferation mechanisms of BURA in oral cancer cells are oxidative-stress-dependent.

### 4.5. Potential Targets

β-ionone is an effective apoptosis inducer in several cancer cell lines derived from gastric, prostate, breast, and bone tissue [55,56,57,58,59]. However, they work through different mechanisms. For example, β-ionone downregulated the extracellular signal-regulated kinase, upregulated p38 and Jun N-terminal kinase [56], and dephosphorylated phosphatidylinositol 3-kinase (PI3K)-AKT levels [57] in gastric cancer cells. β-ionone can activate the prostate-specific G protein-coupled receptor (PSGR) in prostate cancer cells [58], and suppress cyclooxygenase-2 (COX-2) in breast cancer cells [59]. BURA, a β-ionone derivative, contains a carboxylic acid group binding to C-5 of 3-hydroxy-5,6-epoxy-β-ionone. However, the potential targets of BURA remain unclear. This warrants detailed investigation of the changes in the candidate targets of β-ionone in the future.

## 5. Conclusions

The present study, for the first time, confirms that BURA exhibits selective antiproliferation in oral cancer cells with little damage to normal oral cells. Our findings revealed that several kinds of oxidative stress, apoptosis, and DNA damage were upregulated in response to BURA treatment in oral cancer cells. In terms of mechanism, MitoSOX and MMP were dysregulated by BURA. Apoptosis and its caspase signaling were triggered by BURA. γH2AX and 8-OHdG DNA damage was accumulated with BURA treatment in oral cancer cells. Furthermore, these mechanisms were blocked by NAC pretreatment, revealing that oxidative stress plays an important role in regulating antiproliferation by BURA in oral cancer cells. Therefore, our study demonstrated that BURA generates oxidative stress and causes apoptosis and DNA damage that inhibits oral cancer cell proliferation.

## Data Availability

Data are contained within the article.
